# Vehicle Localization Using Crowdsourced Data Collected on Urban Roads

**DOI:** 10.3390/s24175531

**Published:** 2024-08-27

**Authors:** Soohyun Cho, Woojin Chung

**Affiliations:** Department of Mechanical Engineering, Korea University, Seoul 02841, Republic of Korea; csh1360@korea.ac.kr

**Keywords:** vehicle localization, crowdsourced data, simultaneous localization and mapping

## Abstract

Vehicle localization using mounted sensors is an essential technology for various applications, including autonomous vehicles and road mapping. Achieving high positioning accuracy through the fusion of low-cost sensors is a topic of considerable interest. Recently, applications based on crowdsourced data from a large number of vehicles have received significant attention. Equipping standard vehicles with low-cost onboard sensors offers the advantage of collecting data from multiple drives over extensive road networks at a low operational cost. These vehicle trajectories and road observations can be utilized for traffic surveys, road inspections, and mapping. However, data obtained from low-cost devices are likely to be highly inaccurate. On urban roads, unlike highways, complex road structures and GNSS signal obstructions caused by buildings are common. This study proposes a reliable vehicle localization method using a large amount of crowdsourced data collected from urban roads. The proposed localization method is designed with consideration for the high inaccuracy of the data, the complexity of road structures, and the partial use of high-definition (HD) maps that account for environmental changes. The high inaccuracy of sensor data affects the reliability of localization. Therefore, the proposed method includes a reliability assessment of the localized vehicle poses. The performance of the proposed method was evaluated using data collected from buses operating in Seoul, Korea. The data used for the evaluation were collected 18 months after the creation of the HD maps.

## 1. Introduction

Vehicle localization is a fundamental technology essential for applications such as autonomous driving and HD map creation [1]. Localization methods vary depending on the vehicle type, sensor configuration, sensor performance, environment, and specific application [2]. Research on achieving high accuracy and precision in vehicle localization using low-cost sensors remains ongoing [3,4,5,6]. Typically, low-cost sensors are integrated into an onboard device module with embedded processors. This device usually consists of GNSS, an IMU, and cameras, while LiDAR sensors are not utilized.

Localization methods are influenced by the driving environment, which interacts with sensor observations. For example, on highways, the environment is quite open, resulting in minimal GNSS signal interference. The surrounding features are not distinctive, consisting mainly of guardrails and parallel lane markings [7]. In contrast, urban roads often suffer from GNSS signal interference due to surrounding buildings. The lane markings in urban areas reflect complex road structures and intersections, leading to a high density of traffic lights and signs. Environmental characteristics significantly influence the methods for utilizing HD maps and processing sensor data. In this work, we use sensor data from low-cost onboard modules installed in vehicles, collected on urban roads.

Recently, solutions based on crowdsourced data have received significant attention [8,9,10,11,12]. Typically, low-cost sensors are used in vehicles to collect crowdsourced data. When large amounts of data can be obtained cost-effectively, statistical analysis becomes feasible across extensive road networks. Since the statistical analysis is conducted after data acquisition, real-time processing is not required. As a result, the localization method discussed in this study benefits from back-end processing. Vehicle poses derived from crowdsourced data-based localization can be utilized for applications such as HD map updates [8,10,13], road inspections [14], vehicle trajectory analyses [15], and road surveillance [16]. All these applications are influenced by localization errors. The data collected by low-cost sensors generally have high inaccuracy, which negatively impacts localization performance. Research on crowdsourced data-based HD map updates [10] relies on real-time localization by onboard sensors to compare HD maps with observed landmarks. In this context, back-end pose correction is expected to improve map update performance [10].

Offline localization is related to the back end of Simultaneous Localization and Mapping (SLAM). A representative example is SLAM using vehicles equipped with a Mobile Mapping System (MMS) [17]. However, MMS-based methods are not suitable for the problem addressed in this paper, as they typically rely on high-precision data collected from a single vehicle. In contrast, crowdsourced data-based mapping includes a localization scheme that leverages data from multiple vehicles. For instance, graph-based SLAM using sensor data from numerous vehicles on highways was proposed in [18,19]. However, localization methods designed for highway data are challenging to apply directly to urban road scenarios. While successful localization on limited urban roads was demonstrated in [9], our work evaluates localization performance across a broader range of urban roads.

The proposed localization method is designed with consideration for the inaccuracy of crowdsourced data and the environmental characteristics of urban roads. Traffic landmarks recognized by the camera are used for map matching at lane-level HD maps. These lane-level HD maps serve as a layer within the overall HD map for landmarks such as traffic lights, signs, and lane markings. Traffic landmark maps are utilized in vehicle localization through map matching of landmark observations [20,21]. Map matching requires the map and the environment to be identical. Therefore, the proposed method uses the map selectively, based on the reliability assessment of map matching. To take advantage of back-end processing, different localization methods are strategically selected for various sections, rather than relying on sequential tracking. The localization methods in this strategy include bidirectional local tracking based on map matching and factor graph optimization. After localization, a comparison of vehicle poses with odometry measurements is conducted. Additionally, vehicle pose clustering using large amounts of data is employed to detect outliers. As a result, reliable vehicle poses are obtained through localization and diagnostics based on extensive data. The effectiveness of the proposed method was verified through a quantitative evaluation of landmark mapping errors. The evaluation data were collected from numerous buses traveling on various urban roads.

The structure of this paper is as follows. Section 2 discusses localization based on crowdsourced data. Section 3 describes diagnostic methods used for vehicle poses after localization. Section 4 validates the effectiveness of the proposed method through the application of extensive real-world driving data. Finally, Section 5 presents the conclusions.

## 2. Localization Based on Crowdsourced Data

### 2.1. Crowdsourced Data and HD Maps

This section describes the structure of crowdsourced data and the HD maps used for localization. The crowdsourced data in this study are collected from devices equipped with low-cost cameras and GNSS as part of onboard sensor modules. The devices provide refined information processed internally. The information consists of global 3D poses p, odometry related to the 3D poses u, and observations of traffic landmarks z.

The collected data are synchronized on keyframes. Keyframes are generated at regular intervals during travel. In this study, the interval is approximately 5 m. Therefore, the traversal of a single vehicle can be represented as a set of keyframes $\left\{k_{1},\ldots ,k_{N_{k}}\right\}$, where $N_{k}$ is the number of keyframes for a single traversal of the vehicle and is proportional to the travel distance. The information contained in keyframe *i* is expressed as follows:

(1) $$k_{i}=\left\{p_{i},u_{i},z_{i},\Sigma_{p_{i}},\Sigma_{u_{i}},\Sigma_{z_{i}}\right\}$$

In Equation (1), $\Sigma_{p}$, $\Sigma_{u}$, and $\Sigma_{z}$ represent the uncertainty covariance matrices corresponding to p, u, and z, respectively.

Traffic landmarks are recognized by the device’s embedded system through semantic segmentation and are represented relative to the device’s local frame. The 3D positions of traffic landmarks within camera images are obtained by tracking the same landmark during travel. By tracking a single landmark across multiple keyframes, observations are stored only in the keyframe with the lowest observational uncertainty. Traffic signs and lights are represented as 3D points at the centers of features and are defined as discrete landmarks. Lane markings are represented as 3D point sets for lines and are defined as continuous landmarks. In the recognition of dashed lane markings, the point sets for continuous landmarks are modeled to connect the empty spaces continuously, as with solid lines. In summary, the observed sets of discrete and continuous landmarks are denoted as $z_{d}$ and $z_{c}$, respectively, and the observations are expressed as $z=\{z_{d},z_{c}\}$. Similarly, the uncertainty covariance terms are represented as $\Sigma_{z}=\left\{\Sigma_{z_{d}},\Sigma_{z_{c}}\right\}$. The observation uncertainty covariance of continuous landmarks is assigned individually to all points that constitute the landmark.

**Figure 1:**
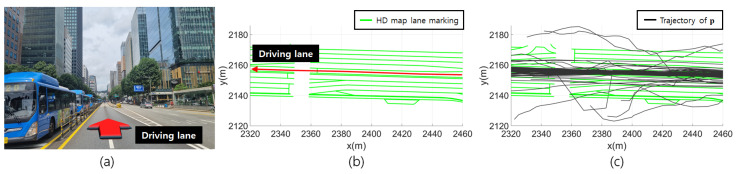
Localization error in p across multiple traversals. (**a**) Urban road image. (**b**) HD map with ground-truth driving lane corresponding to the driving lane in (**a**). (**c**) Trajectory of p for 172 traversals.

The accuracy and precision of p estimated in real time by onboard devices are not guaranteed. Figure 1 visually illustrates the localization errors of p across multiple traversals on a specific urban road. As shown in Figure 1a, there are buildings surrounding the roads that obstruct GNSS signals. A comparison between Figure 1b and Figure 1c reveals that p frequently deviates from the ground-truth driving lane. There is a high probability that the significant bias errors in p are due to GNSS multipath errors. Such errors are difficult to represent with $\Sigma_{p}$. Therefore, it is important to correct p to account for data uncertainty and environmental characteristics.

The proposed method partially utilizes HD maps. To match the observed landmarks in crowdsourced data, feature maps corresponding to the types of observed landmarks in the HD map are used. The feature maps are categorized into discrete landmarks of 3D points $M_{d}$ and continuous landmarks of 3D point sets $M_{c}$, identical to the classification of observed landmark types. Therefore, the HD map M used in the proposed method can be expressed as follows:

(2) $$M=\{M_{d},M_{c}\}$$

The structure of the crowdsourced data utilized in this study is atypical. The crowdsourced data are identical to those used in previous research [10] and similar to those used in [8].

### 2.2. Bidirectional Local Tracking with Map Matching

This section introduces map matching-based localization for a partial set of keyframes in a single traversal. The proposed localization is a process to correct p, which is subject to high inaccuracies. Pose correction relative to the map frame can utilize landmarks such as traffic signs, lights, and lane markings in the HD map or links to the driving lane. However, there are cases where the lateral error of p is larger than the interval between adjacent lanes. In such cases, it becomes difficult to estimate the driven lane due to the similar geometric characteristics of the lane markings. Therefore, instead of uniformly correcting all keyframes in the same manner, an appropriate localization method is applied to each keyframe according to observations and map-matching states.

To achieve reliable localization within the map frame, accurate matching of observed landmarks is essential. ICP-based nearest-point matching can be utilized to find correspondences for landmarks [22]. However, nearest-point matching can cause p to converge on the incorrect lane. Therefore, we begin by identifying a single keyframe where reliable map matching is possible. To estimate a stable 3D pose through map matching, we focus on a keyframe where at least one discrete landmark and two continuous landmarks are recognized. If only continuous landmarks are recognized, correcting the direction of travel becomes difficult. Since traffic signs and lights are located at specific points along the road, the number of keyframes that satisfy the landmark observation condition is limited.

To find accurate landmark correspondences based on the inaccurate p, costs are assigned and evaluated for all discrete landmark correspondences within a certain range. Considering the inaccuracy of p, HD map landmarks of the same type as the observed discrete landmark within a distance $d_{g}$ are selected as matching candidates. The distance $d_{g}$ is empirically set based on the maximum positional error of p. The matching candidates $M_{cand}\left(z_{d}\right)$ for a single observed discrete landmark $z_{d}\;\left(z_{d}\in z_{d}\right)$ in keyframe *i* are expressed as follows:

(3) $$M_{cand}\left(z_{d}\right)=\{m_{d}\left|\;|\right|p_{i}⊕z_{d}-m_{d}||<d_{g},m_{d}\in M_{d}\}$$

In Equation (3), $m_{d}$ represents a discrete landmark included in the HD map. The operator ⊕ denotes pose composition. Each element of the matching candidate $M_{cand}\left(z_{d}\right)$ is evaluated by a matching cost. The matching cost is calculated based on the nearest-point match for landmarks after an initial translation $t_{0}$ by candidate correspondences. The initial translation $t_{0}$ represents the positional error between $z_{d}$ and the candidate in the map frame. $p_{i}$ becomes $p_{i}^{-}$ through the translation by $t_{0}$ and is expressed as follows:

(4) $$t_{0}=p_{i}⊕z_{d}-M_{cand}\left(z_{d}\right)$$

(5) $$p_{i}^{-}=p_{i}⊕\left[R_{0},t_{0}\right]\;\left(R_{0}=I_{3}\right)$$

In Equation (5), $R_{0}$ is the identity matrix for three-dimensional rotation. The correspondence of continuous landmarks and other discrete landmarks depends on the nearest-point matching at $p_{i}^{-}$. From this correspondence, error vectors for discrete landmarks $e_{d}=\left(p_{i}^{-}⊕z_{d}\right)-m_{d}$ and continuous landmarks $e_{c}=\left(p_{i}^{-}⊕z_{c}\right)-m_{c}$ can be derived within the map frame. The matching cost of landmarks $f_{z}\left(p_{i}^{-}\right)$ is expressed as the weighted average of the Mahalanobis distances, as follows:

(6) $$f_{z}\left(p_{i}^{-}\right)=\frac{w_{d}}{n_{d}}\sum e_{d}^{T}{\left(R_{i}^{-}\Sigma_{z_{d}}{\left(R_{i}^{-}\right)}^{T}\right)}^{-1}e_{d}+\frac{w_{c}}{n_{c}}\sum e_{c}^{T}{\left(R_{i}^{-}\Sigma_{z_{c}}{\left(R_{i}^{-}\right)}^{T}\right)}^{-1}e_{c}$$

In Equation (6), $R_{i}^{-}$, $w_{d}$, $w_{c}$, $n_{d}$, and $n_{c}$ represent the rotation matrix SO(3) of $p_{i}^{-}$, the weights of discrete and continuous landmarks, the number of matched discrete landmarks, and the number of points in matched continuous landmarks, respectively. The matching cost $f_{z}\left(p_{i}^{-}\right)$ is minimized through iterative optimization based on the Levenberg–Marquardt method [23]. After optimization, the optimal vehicle pose $p_{i}^{cand}$ is estimated from the candidate correspondences.

For all observed discrete landmarks, matching candidates are selected, and the matching costs $f_{z}\left(p_{i}^{cand}\right)$ after optimization for each candidate are derived. If errors are minimized by incorrect landmark correspondences, $f_{z}\left(p_{i}^{cand}\right)$ is typically abnormally high. To assess the validity of each candidate, the maximum value of $f_{z}\left(p_{i}^{cand}\right)$ for correct matching is empirically set as $f_{z,valid}$. Therefore, a candidate with an $f_{z}\left(p_{i}^{cand}\right)$ less than $f_{z,valid}$ is considered valid. However, the number of candidates that satisfy the validity criterion varies depending on the conditions. If no valid candidates are found, it is likely due to misdetected landmarks, high measurement uncertainty, or the detection of newly created landmarks not present on the map. Conversely, if more than two valid candidates exist, it is likely due to the similarity of road structures between adjacent lanes. In such cases, the reliability of candidate matching is low. Therefore, the optimal pose $p_{i}^{*}$ is determined only when a single valid matching candidate exists. $p_{i}^{*}$ is expressed by the following equation, which minimizes the matching cost:

(7) $$p_{i}^{*}=\underset{p_{i}^{cand}}{\mathrm{argmin}}\left\{f_{z}\left(p_{i}^{cand}\right)\right\}$$

If reliable localization based on discrete landmark matching candidates is achieved at keyframe *i*, reliable pose estimation using odometry is possible for the adjacent areas of keyframe *i*. By taking advantage of the offline system, odometry-based estimation can be performed in both the forward and reverse directions of the vehicle. The predicted poses of adjacent keyframes, estimated based on odometry from keyframe *i*, can be expressed by the following equation:

(8) $$p_{i+1}^{-}=p_{i}^{*}⊕u_{i}$$

(9) $$p_{i-1}^{-}=p_{i}^{*}⊕u_{i-1}^{-1}$$

Equation (8) represents the estimation in the direction of the vehicle’s movement. In contrast, Equation (9) indicates the estimation in the opposite direction of the vehicle. The term $u_{i-1}^{-1}$ represents the inverse transformation of $u_{i-1}$. From the odometry-based predicted pose $p^{-}$, the optimal pose is derived through corrections that include map matching. The correspondences for map matching are determined by nearest-point matching with respect to the predicted pose. The cost based on the landmark matching error follows Equation (6). Additionally, the odometry-based predicted position is incorporated into the cost through point-to-point matching. The odometry-based predicted position serves as a constraint in the direction of the vehicle’s movement. The odometry-based position error vector $e_{u}$ is expressed as follows:

(10) $$e_{u}={\left(p_{i+1}^{-}⊕\Delta T\right)}_{t}-{\left(p_{i+1}^{-}\right)}_{t}$$

In Equation (10), ${\left(\cdot \right)}_{t}$ refers to the selection of the translation term from either the pose vector or the covariance matrix. ΔT is the transformation that corrects the predicted pose $p_{i+1}^{-}$ in the iterative optimization process of reducing the cost function. Consequently, the local tracking cost function $f_{L}\left(p_{i+1}^{-}\right)$ is expressed as follows:

(11) $$f_{L}\left(p_{i+1}^{-}\right)=f_{z}\left(p_{i+1}^{-}\right)+w_{u}e_{u}^{T}{\left(R_{i}^{-}{\left(\Sigma_{u_{i}}\right)}_{t}{\left(R_{i}^{-}\right)}^{T}\right)}^{-1}e_{u}$$

In Equation (11), $R_{i}^{-}$ and $w_{u}$ represent the rotation matrix SO(3) of $p_{i}^{-}$ and the weight for the odometry-based predicted position, respectively. The cost function $f_{L}\left(p_{i+1}^{-}\right)$ is minimized using the Levenberg–Marquardt method [23], resulting in the optimal pose $p_{i+1}^{*}$. For local tracking in the opposite direction of the vehicle’s movement, in Equations (10) and (11), $p_{i+1}^{-}$ and $\Sigma_{u_{i}}$ are replaced with $p_{i-1}^{-}$ and $\Sigma_{u_{i-1}^{-1}}$, respectively.

The vehicle poses of adjacent keyframes in both directions are estimated sequentially from the estimated keyframe *i* in Equation (7). However, localization confidence can be reduced by factors such as a lack of observed landmarks, high measurement uncertainties, and environmental changes. In these cases, confidence can be assessed by the number of observed continuous landmarks and the cost after optimization $f_{L}\left(p^{*}\right)$ in local tracking. For stable 3D pose estimation, at least two continuous landmarks are recognized, and the cost $f_{L}\left(p^{*}\right)$ is lower than the empirically set threshold $f_{L,valid}$. However, the conditions required for reliable localization may not be met under temporary occlusions by other vehicles or short-term environmental changes. To maximize the number of keyframes estimated through local tracking, temporary low-confidence pose estimations are replaced with odometry-based predictions. If localization confidence is not maintained for a certain number of consecutive keyframes, local tracking is considered a failure.

Due to the characteristics of the road structure, only parallel lane markings are observed in straight road sections without traffic signs or lights. In this case, even if $f_{L}\left(p^{*}\right)$ remains consistently low during local tracking over successive keyframes, drift error in the direction of the vehicle’s movement accumulates. Therefore, if drift error correction is not achieved through map matching of discrete landmarks, local tracking is terminated based on the magnitude of the propagating position uncertainty covariance matrix from odometry. The odometry-based propagated position uncertainty covariance matrix $\Sigma_{i+k}$ for consecutive keyframes from *i* to i+k is expressed as follows:

(12) $$\Sigma_{i+k}=\Sigma_{i+k-1}+R_{i+k-1}^{-}{\left(\Sigma_{u_{i+k-1}}\right)}_{t}{\left(R_{i+k-1}^{-}\right)}^{T}\;\;\;\left(\Sigma_{i}=O_{3\times 3}\right)$$

According to Equation (12), local tracking at keyframe *i* includes map matching with discrete landmarks. The decision to terminate local tracking is based on the sum of the diagonal elements of the propagated uncertainty covariance matrix, $trace(\Sigma_{i+k})$. Bidirectional local tracking from a single reference keyframe generates two termination keyframes. The keyframes between these two termination keyframes can be considered as having reliable localization with map matching applied. By repeatedly applying bidirectional local tracking to the remaining unestimated keyframes, keyframes with reliable map matching-based estimations can be separated from the entire keyframe set.

### 2.3. Local Pose Graph Optimization

This section addresses the localization of keyframes that are not estimated by the bidirectional local tracking process. Since unestimated keyframes are likely to be difficult for map matching, we applied pose graph-based optimization without map matching. The keyframes successfully estimated during bidirectional local tracking can be used as fixed nodes in the pose graph. Therefore, instead of generating a pose graph for the entire set of keyframes from a single traversal, multiple local pose graphs were individually created and optimized based on the fixed nodes.

**Figure 2:**
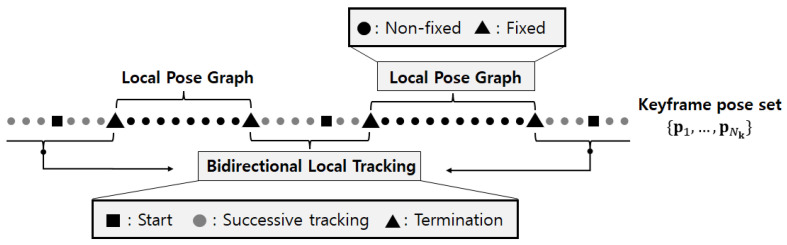
Localization method for keyframes in a single traversal.

Figure 2 shows an example of the range within the keyframe set of a single traversal where local pose graphs are generated. Two local pose graphs are created based on the termination keyframes of bidirectional local tracking. The nodes of the local pose graph include two fixed nodes and p. Given the interval of keyframes from *i* to i+n, the initial node set of the local pose graph is denoted as $X=\{x_{i},x_{i+1},\ldots ,x_{i+n-1},x_{i+n}\}$. It can be expressed as follows:

(13) $$X=\{p_{i}^{*},p_{i+1},\ldots ,p_{i+n-1},p_{i+n}^{*}\}$$

In Equation (13), $x_{i}$ and $x_{i+n}$ correspond to the fixed nodes $p_{i}^{*}$ and $p_{i+n}^{*}$, respectively. The edge constraints of the local pose graph include the error between the odometry measurement and the transformation of successive nodes. The edge constraint for frame *j* to j+1 is represented as $e_{j,j+1}$. Therefore, the error vector based on the odometry measurement $e_{j,j+1}$ and the corresponding information matrix $\Omega_{j,j+1}$ is expressed as follows:

(14) $$e_{j,j+1}=u_{j}^{-1}⊕\left(x_{j}^{-1}⊕x_{j+1}\right)\;\;\left(i\leq j<i+n\right)$$

(15) $$\Omega_{j,j+1}={\left(\Sigma_{u_{j}}\right)}^{-1}$$

In Equation (14), the expression for the error vector follows that in [24]. The odometry-based constraint smooths the keyframe pose between two fixed nodes through pose graph optimization. When optimizing the pose graph using only odometry-based constraints, drift error accumulates in the keyframe poses further from the fixed nodes as the travel distance between them increases. By adding p as an edge constraint for the global position, the drift errors can be corrected. Considering the positional inaccuracy of p, constraints for the global position are generated intermittently. As shown in Equation (12), when the uncertainty covariance of odometry-based drift errors propagated from the fixed nodes becomes larger than the inaccuracy of p, a global position constraint is added. When a global position constraint is generated at keyframe *k*, the error vector is expressed as a unary edge as follows:

(16) $$e_{k,k}={\left(x_{k}\right)}_{t}-{\left(p_{k}\right)}_{t}$$

The information matrix $\Omega_{k,k}$, corresponding to the error vector $e_{k,k}$, is determined based on the positional inaccuracy of p. The standard deviation of the positional uncertainty $\sigma_{p}$, is determined empirically by estimating the maximum positional error between the ground truth and p. Therefore, $\Omega_{k,k}$ is expressed as follows:

(17) $$\Omega_{k,k}=\left(\begin{array}{ccc} {\left(\sigma_{p}^{2}\right)}^{-1} & 0 & 0\\ 0 & {\left(\sigma_{p}^{2}\right)}^{-1} & 0\\ 0 & 0 & 0 \end{array}\right)$$

Combining the constraints from Equations (14)–(17), the cost function of the pose graph is expressed as follows:

(18) $$F(X)=\sum_{j}e_{j,j+1}^{T}\Omega_{j,j+1}e_{j,j+1}+\sum_{k}e_{k,k}^{T}\Omega_{k,k}e_{k,k}$$

The cost function F(X) is minimized using pose graph optimization based on the g2o library [25]. After optimization, the pose graph nodes $X^{*}$ become the optimized keyframe poses $p^{*}$ for the local pose graph area.

## 3. Localization Diagnosis

### 3.1. Odometry-Based Keyframe Pose Diagnosis

The proposed localization method includes a process for assessing the reliability of localization. However, due to the similarity of road structures, bidirectional local tracking can be performed in the incorrect lane. In this case, the local pose graph optimization is affected by significant errors in the fixed nodes. Therefore, we diagnose $p^{*}$ by comparing the transformation between the keyframe poses after localization and the odometry.

The error vector $e_{u}^{*}$ between consecutive keyframes *i* and i+1 after localization is identical to the odometry constraint in the pose graph, as shown in Equation (14).

(19) $$e_{u}^{*}=u_{i}^{-1}⊕\left({\left(p_{i}^{*}\right)}^{-1}⊕p_{i+1}^{*}\right)$$

To diagnose the localization over a certain distance within a keyframe segment, we evaluate the average error for *n* consecutive keyframes. The exclusion condition for the evaluated *n* keyframes is expressed as follows:

(20) $$\frac{1}{n}\sum^{n}{\left(e_{u}^{*}\right)}^{T}{\left(\Sigma_{u}\right)}^{-1}e_{u}^{*}>d_{odom}$$

The threshold value $d_{odom}$ in Equation (20) is determined empirically. Odometry-based pose diagnosis allows for the exclusion of keyframe sets that exhibit excessive differences compared to odometry-based predictions.

### 3.2. Pose Clustering with Outlier Filtering

In this part, keyframe outliers are excluded through the clustering of keyframe poses. If the ratio of traversals between adjacent lanes differs significantly, the data from lanes with fewer traversals are less likely to represent the majority and are more likely to be considered outliers.

Keyframe pose clustering considers both the position and heading direction of the keyframes. The proposed method utilizes an approach that adds a vehicle heading direction metric to DBSCAN-based vehicle position clustering [26]. In the clustering of positions, the distance between clusters is set to ensure that clusters from different lanes are separated, considering the standard lane interval. Clustering based on the heading direction groups vehicles traveling in the same direction and distinguishes between adjacent lanes with opposite directions of travel or different directions at intersections.

The number of keyframes included in a cluster varies depending on the number of traversals and the presence of outliers. Evaluation for a single cluster is conducted by comparing it with clusters in adjacent areas. If the number of keyframes in the cluster is significantly smaller than in surrounding clusters, the cluster is considered an outlier. To statistically detect outliers, we utilize the MAD (Median Absolute Deviation) filter [27]. The MAD filter identifies data points that deviate significantly from the median as outliers. In our problem, only outliers with a smaller number of keyframes are selected. The scope of the MAD filter should account for the varying number of traversals for each road and lane marking. For a single cluster $c_{i}$, the adjacent cluster set $C_{MAD,i}$ for comparison is determined by the average positional and heading direction differences between clusters. $C_{MAD,i}$ is expressed as follows:

(21) $$C_{MAD,i}=\left\{c\;|\;|\right|C_{p}\left(c\right)-C_{p}\left(c_{i}\right)||<d_{p},||C_{h}\left(c-C_{h}\left(c_{i}\right)\right)||<d_{h}\}$$

In Equation (21), $C_{p}\left(c\right)$ and $C_{h}\left(c\right)$ represent the average position and average heading direction of the keyframes contained in cluster c. $d_{p}$ and $d_{h}$ are the position and heading direction thresholds of the adjacent clusters. When the MAD filter is applied to $C_{MAD,i}$, the outlier status of each element is determined. To increase the reliability of outlier detection, $C_{MAD}$ is generated for each cluster, and the outlier status is redundantly evaluated across all clusters. Subsequently, a single cluster receives $N_{evalation}$ outlier evaluations from adjacent clusters. If the outlier detection rate is high across these multiple evaluations, the cluster is excluded. The condition for a single cluster to be classified as an outlier is as follows:

(22) $$\frac{N_{outlier}}{N_{evaluation}}>r_{outlier}$$

In Equation (22), $N_{outlier}$ and $r_{outlier}$ represent the number of outlier determinations and the criterion for the cluster exclusion rate, respectively. The outlier status is evaluated for all clusters. Finally, the keyframe poses contained in the clusters that have not been excluded are utilized.

## 4. Evaluation

For the evaluation, we used bus driving data from roads in Seoul, Korea. The evaluation data were collected on specific sections of the roads over two days, 18 months after the HD map was created.

**Figure 3:**
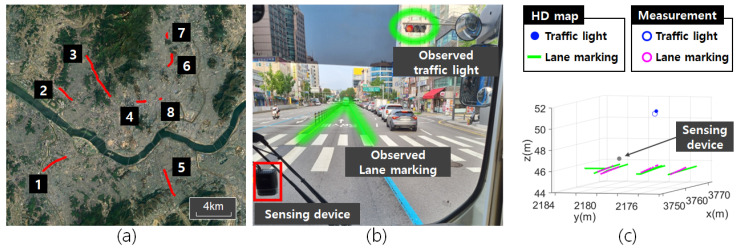
Visualization of data acquisition roads and sensor observations. (**a**) Satellite view of data acquisition roads. (**b**) The sensing device mounted on the vehicle and the observed traffic landmarks. (**c**) HD map and landmark measurements associated with a single keyframe.

Figure 3a shows a satellite image of the urban area where the evaluation dataset was collected. The data were collected from eight separate roads, with a total length of approximately 35.1 km. Multiple driving datasets were obtained from these roads, with a total driving distance of 4214 km. Figure 3b,c show examples of traffic landmarks observed by sensing devices mounted on the vehicle.

To verify the validity of the proposed localization method, we assessed the consistency between the ground-truth path and the estimated vehicle positions and driving directions. A keyframe pose estimation was considered successful if the estimated keyframe position was closest to the link of the ground-truth driving path in the HD map and the driving directions were matched. When environmental changes occurred, the HD map link was updated to reflect the conditions at the time of data acquisition and then used for evaluation. The maximum valid matching distance criterion between the keyframe and the HD map link was set to 1.5 m in accordance with lane marking width standards. For quantitative evaluation, the ratio of keyframes with successfully estimated driving paths was compared based on whether the proposed method was applied.

Table 1 presents the quantitative analysis results for the eight areas shown in Figure 3a. “Rejection” in the table indicates the ratio of keyframes excluded by the proposed method. The rejection rates varied by area, ranging from 48% to 69%. The variation in rejection rates was due to the challenges of localization posed by different road environments. The results indicate that even in the worst-case scenario, more than 30% of the data were still utilized. “Raw data” and “Proposed” in the table represent the valid ratios of keyframe poses for p and the proposed method, respectively. The keyframe validity ratio for the proposed method showed no significant variation across different environments, maintaining approximately 90%. By applying the proposed method, the keyframe validity ratio increased in all areas. Overall, the ratio of invalid keyframes in the raw data was approximately three times higher compared to the proposed method. Invalid keyframe poses are likely to cause performance degradation when used in solutions such as HD map creation or environmental change detection.

**Figure 4:**
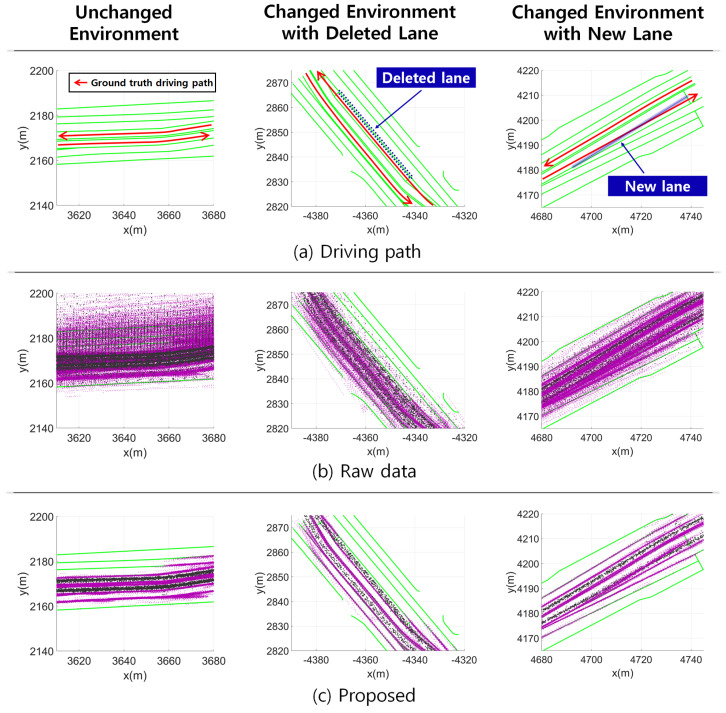
Qualitative results of localization and mapping with environmental changes. (**a**) Ground-truth driving path with HD map lane markings. (**b**) Keyframe positions and continuous landmarks from raw data across multiple traversals. (**c**) The corresponding figure for (**b**) after applying the proposed method. Black dots represent keyframe positions, and purple dots represent continuous landmarks based on the keyframe pose.

Figure 4 shows the qualitative evaluation results of localization for the road without environmental changes, the road with a newly created lane marking, and the road with a deleted lane marking. Environmental changes are represented by the creation of new landmarks not present on the map or the removal of existing landmarks from the map. By comparing Figure 4b,c, it is evident that the estimated poses mostly matched the ground-truth driving path when the proposed method was applied. The proposed method partially utilized map matching and excluded unreliable localization results. Nevertheless, as shown in Figure 4c, the mapped continuous landmarks correctly reflect the environmental changes.

Localization performance is closely related to the accuracy of mapping. To quantitatively evaluate localization, we assessed the mapping accuracy and precision of landmarks. The mapping of landmarks observed from multiple driving datasets and their correspondence with the HD map was conducted using the method described in previous research [10]. In [10], the positions of mapped discrete landmarks were estimated using Maximum A Posteriori (MAP) [28], considering the observation uncertainty for the same landmarks recognized from driving datasets. Similarly, the mapped continuous landmark is a point array obtained by clustering the lane markings detected during multiple traversals and the HD map, followed by fitting based on a Bézier curve [29].

Table 2 presents the results of the mapping error evaluation for the 198 traffic lights and signs within the sensor’s field of view. By applying the proposed method, both the accuracy and precision of the mapping were improved. Precision is measured as the average distance error between the mapped landmark and the landmark measurements.

**Figure 5:**
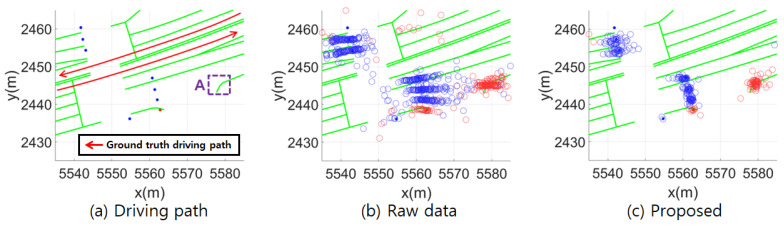
Comparison of discrete landmark mapping results between the raw data and the proposed method. Filled red and blue circles represent traffic signs and traffic lights on the HD map, respectively. Correspondingly, the empty red and blue circles represent the mapped observed landmarks.

Figure 5 shows the mapping results of the discrete landmarks. In Figure 5b, the discrete landmarks mapped by multiple vehicles display high variance with respect to the driving direction. In contrast, Figure 5c shows lower variance along with fewer outliers. Region “A” in (a) indicates a newly created traffic sign that was not included in the HD map. Figure 5c shows the observed discrete landmarks corresponding to the region, and the variance is similar to that of other discrete landmarks.

The continuous landmarks were evaluated in the same manner as the discrete landmarks. As shown in Table 3, the mapping accuracy and precision improved when using the proposed method compared to the raw data. Precision is calculated as the average distance error between the mapped continuous landmark obtained by fitting and the measured continuous landmark points.

**Figure 6:**
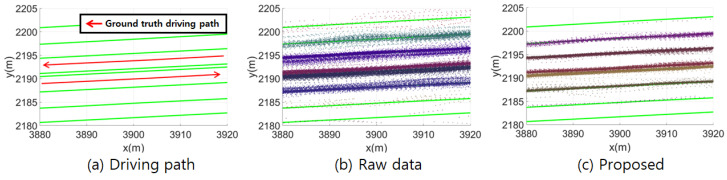
Comparison of continuous landmark mapping results between the raw data and the proposed method. Points of the same color represent continuous landmarks clustered in the same HD map lane marking.

Figure 6 shows the mapping results of the continuous landmarks. Comparing Figure 6b and Figure 6c, the thickness of the clustered lane marking measurement points for a single HD map lane marking is thinner with the proposed method than with the raw data. A thinner distribution of clustered continuous landmark points indicates a smaller lateral error in the keyframe poses. Therefore, using the vehicle pose estimated by the proposed method improves the accuracy and precision of landmark mapping. In other words, the mapping results validate the effectiveness of the proposed localization method.

## 5. Conclusions

In this paper, we address offline vehicle localization using a large amount of crowdsourced data. The proposed localization method is designed with consideration for the inaccuracies in crowdsourced data and the structural characteristics of urban road traffic landmarks. To account for these characteristics, we strategically apply map matching and factor graph optimization in the back end based on specific conditions. Leveraging the advantage of large datasets, inaccurate localized keyframes are excluded through a diagnostic process. This diagnostic method involves comparing the estimated keyframe poses with odometry and filtering outliers through keyframe pose clustering using a large amount of driving data. Consequently, reliable localized keyframe poses can be obtained from data collected by multiple vehicles.

The proposed method was validated using a large amount of crowdsourced data collected on urban roads. Localization was evaluated by verifying the driving path and comparing landmark mapping errors. The reliably estimated poses from multiple vehicles are expected to be applicable for map updates, vehicle trajectory estimation, mapping, and road infrastructure management.

## Figures and Tables

**Table 1:** Comparison of valid keyframe ratios resulting from the use of the proposed method in each evaluation area.

Area	Length of Link (km)	Driving Distance (km)	Rejection (%)	Raw Data (%)	Proposed (%)
1	5.8	340.8	63.8	72.0	94.5
2	4.3	338.4	67.5	63.9	90.2
3	10.6	1289.8	52.8	74.4	91.8
4	2.2	945.7	48.4	75.0	88.3
5	6.0	763.3	69.4	54.4	89.6
6	3.3	386.0	51.1	75.6	92.7
7	2.2	71.8	57.5	72.2	98.3
8	0.7	77.7	62.8	75.1	91.8
**Overall**	**35.1**	**4213.5**	**57.0**	**69.9**	**90.9**

**Table 2:** Evaluation of discrete landmark mapping: 198 traffic lights and signs were used.

Method	Accuracy (m)	Precision (m)
Raw data (**p**)	1.05	1.46
Proposed	0.53	0.78

**Table 3:** Evaluation of continuous landmark mapping: The total length of lane markings is 122.6 km.

Method	Accuracy (m)	Precision (m)
Raw data (**p**)	0.29	0.39
Proposed	0.21	0.21

## Data Availability

The original contributions presented in the study are included in the article, and further inquiries can be directed to the corresponding author.
